# Whole genome sequencing diagnostic yield for paediatric patients with suspected genetic disorders: systematic review, meta-analysis, and GRADE assessment

**DOI:** 10.1186/s13690-023-01112-4

**Published:** 2023-05-25

**Authors:** Mario Cesare Nurchis, Gerardo Altamura, Maria Teresa Riccardi, Francesca Clementina Radio, Giovanni Chillemi, Enrico Silvio Bertini, Jacopo Garlasco, Marco Tartaglia, Bruno Dallapiccola, Gianfranco Damiani

**Affiliations:** 1 Department of Woman and Child Health and Public Health, Fondazione Policlinico Universitario A. Gemelli IRCCS, 00168 Rome, Italy; 2 School of Economics, Università Cattolica del Sacro Cuore, 00168 Rome, Italy; 3 Department of Health Sciences and Public Health, Section of Hygiene, Università Cattolica del Sacro Cuore, Largo Francesco Vito 1, 00168 Rome, Italy; 4 Genetics and Rare Diseases Research Division, Ospedale Pediatrico Bambino Gesù IRCCS, 00146 Rome, Italy; 5 Department for Innovation in Biological Agro-Food and Forest Systems (DIBAF), University of Tuscia, 01100 Viterbo, Italy; 6 Institute of Biomembranes, Bioenergetics and Molecular Biotechnologies, Centro Nazionale Delle Ricerche, 70126 Bari, Italy; 7 Department of Public Health Sciences and Paediatrics, University of Turin, 10126 Turin, Italy

**Keywords:** NGS, Paediatrics, Diagnostic yield, Public health, Health policy

## Abstract

**Background:**

About 80% of the roughly 7,000 known rare diseases are single gene disorders, about 85% of which are ultra-rare, affecting less than one in one million individuals. NGS technologies, in particular whole genome sequencing (WGS) in paediatric patients suffering from severe disorders of likely genetic origin improve the diagnostic yield allowing targeted, effective care and management. The aim of this study is to perform a systematic review and meta-analysis to assess the effectiveness of WGS, with respect to whole exome sequencing (WES) and/or usual care, for the diagnosis of suspected genetic disorders among the paediatric population.

**Methods:**

A systematic review of the literature was conducted querying relevant electronic databases, including MEDLINE, EMBASE, ISI Web of Science, and Scopus from January 2010 to June 2022. A random-effect meta-analysis was run to inspect the diagnostic yield of different techniques. A network meta-analysis was also performed to directly assess the comparison between WGS and WES.

**Results:**

Of the 4,927 initially retrieved articles, thirty-nine met the inclusion criteria. Overall results highlighted a significantly higher pooled diagnostic yield for WGS, 38.6% (95% CI: [32.6 – 45.0]), in respect to WES, 37.8% (95% CI: [32.9 – 42.9]) and usual care, 7.8% (95% CI: [4.4 – 13.2]). The meta-regression output suggested a higher diagnostic yield of the WGS compared to WES after controlling for the type of disease (monogenic vs non-monogenic), with a tendency to better diagnostic performances for Mendelian diseases. The network meta-analysis showed a higher diagnostic yield for WGS compared to WES (OR = 1.54, 95%CI: [1.11 – 2.12]).

**Conclusions:**

Although whole genome sequencing for the paediatric population with suspected genetic disorders provided an accurate and early genetic diagnosis in a high proportion of cases, further research is needed for evaluating costs, effectiveness, and cost-effectiveness of WGS and achieving an informed decision-making process.

**Trial Registration:**

This systematic review has not been registered.

**Supplementary Information:**

The online version contains supplementary material available at 10.1186/s13690-023-01112-4.

## Introduction

Online Mendelian Inheritance in Man (OMIM), an updated catalogue of human genes and genetic phenotypes, contains over 16,000 genes [1, 2], and more than 9,300 Mendelian phenotypes, including more than 6,000 with a known molecular defect. In addition, there are more than 1,500 confirmed Mendelian phenotype or a phenotypic locus with an unknown molecular basis and more than 1,700 additional phenotypes of suspected Mendelian origin [3]. According to Orphanet [4] there are roughly 7,000 rare diseases (RD), 80% of which are thought to have a genetic cause, the majority of which are Mendelian/monogenic disorders [5, 6].

Non-monogenic genetic diseases, also known as complex genetic diseases, are conversely caused by a combination of genetic, environmental, and lifestyle factors. Unlike monogenic diseases that are caused by a single gene mutation, non-monogenic diseases involve multiple genes, each contributing a small effect, as well as environmental and lifestyle factors. According to the European definition, rare diseases are life-threatening or chronically debilitating conditions with a prevalence of less than one case in 2,000 individuals, while the US figure is less than one case in 1,500 people [7, 8]. Although rare individually, these disorders affect 264–446 million of people worldwide, and 17.8–30.3 million in Europe [7, 9]. About 50 to 60% of RDs affect children, 12% of which are congenital and 42% have an onset in the first two years of life [9, 10]. Patients suffering from RD share similar needs, suffer diagnostic delay, uncertainty in genetic counselling, and lack of proper clinical management and care, since an effective treatment is available only for about 400 diseases. This is also due to failure in the identification of the molecular defects underlying a large number of these diseases. Genetic testing confirms or rules out a suspected genetic condition, is diagnostic in a proportion of clinically unsolved cases and determines the individual chance of developing or passing on a genetic disorder. Over the past decade, the development of next generation sequencing (NGS) technologies and bioinformatic pipelines to manage and analyse genomic data, jointly with an impressive reduction of sequencing costs, have led to a widespread implementation of genomic sequencing, most often whole exome sequencing (WES). These tools, which can identify the molecular defect causing Mendelian disorders [11], have shown to be effective and sustainable in genomic medicine. As a powerful tool, genomic medicine has the potential to improve outcomes and reduce costs in primary care settings [12]. The application of whole genome sequencing (WGS) and the whole exome sequencing (WES) in new-borns and children suffering from a severe disorder of likely genetic origin is expected to improve targeted, effective care and management [13, 14].

WGS and WES are increasingly used for diagnostic purposes on critically ill infants and children admitted to Neonatal Intensive Care Units (NICU) and Paediatric Intensive Care Units (PICU) with a suspected genetic disorder [14–16]. Traditional genetic testing allows to reach the diagnosis in around 20% of cases [14]. Thus, acutely ill neonates with suspected genetic diseases are often discharged or deceased before diagnosis. As a result, NICU treatment of genetic diseases is usually empirical, may lack efficacy, may be inappropriate, or even may cause adverse effects [17].

Currently, whole exome sequencing (WES) is more commonly used globally than whole genome sequencing (WGS) due to its easier data storage and processing [18], as well as its cost-saving benefits [19]. Nevertheless, despite the widespread adoption of whole exome sequencing (WES), previous research has demonstrated that whole genome sequencing (WGS) has the potential to yield a greater number of diagnoses than WES both in undiagnosed adults and suspected genetic diseases of the newborn. Particularly, over a large number of studies, the diagnostic yield attained by WES ranges between 25 and 50% while the WGS diagnostic yield is about 40–60% [20, 21]. Furthermore, a recent systematic review and meta-analysis showed a greater diagnostic yield for WGS (0.41, 95% CI 0.34–0.48, I2 = 44%) compared to WES (0.36, 95% CI 0.33–0.40, I2 = 83%), although not statistically significant, and usual care (UC) (0.10, 95% CI 0.08–0.12, I2 = 81%) [22]. In addition, another systematic review reported a diagnostic yield ranging from 3 to 79% for WES and between 17 and 73% for WGS [23].

Recent studies have also supported the clinical utility of WGS, compared to standard testing, in NICU highlighting a higher diagnostic yield, a sharp increase in changes in clinical management, and shortening of the time to diagnosis thanks to the PCR-free WGS approach[24]. Therefore, a wider use of WGS could change acute management and life outcomes in children with chronic diseases using stratified therapeutics [14].

Marshall et al. [25]. In addition, the translation of WGS into clinical settings has been hindered by the lack of access to technology, complex infrastructure, and expert personnel. At present, in a context of limited healthcare resources, it is necessary to retrieve evidence on how to integrate the WGS technology in the diagnostics, fulfilling both the criteria of clinical utility and cost-effectiveness [26].

The aim of this study was to perform a systematic review and meta-analysis to assess the effectiveness of WGS, with respect to WES and/or UC, for the diagnosis of suspected genetic disorders among the paediatric population.

## Materials and methods

### Search strategy and selection criteria

A systematic review of the literature was conducted querying relevant electronic databases, including MEDLINE, EMBASE, ISI Web of Science, and Scopus from January 2010 to June 2022 in order to retrieve peer-reviewed articles. The Population, Intervention, Comparison, Outcome (PICO) [27] framework was adopted to formulate the following research question: “*Is implementing WGS for the care of the paediatric population effective?*”. A comprehensive search strategy was created and implemented according to the Preferred Reporting Items for Systematic Reviews and Meta-Analyses (PRISMA) [28] checklist. First, controlled descriptors and the relative key words were identified and verified in each scientific database. Afterwards, a Boolean search string, combining Medical Subject Headings (MeSH) and free-text words, such as “new born”, “infant”, “paediatric”, “paediatric”, “child”, “next-generation sequencing”, “whole genome sequencing”, “whole exome sequencing”, “genomic testing”, “panel test”, “diagnostic yield”, “effectiveness”, “appropriateness”, “efficacy”, “clinical efficacy”, “NICU”, “PICU”, “emergency”, was used. Full search strings for each database are detailed in the Supplementary Material. The search was completed by hand search in order to identify missing articles (i.e., snowball searching). Additional relevant articles were found by analysing bibliographic citations. The inclusion criteria for this systematic review were defined as follows: paediatric patients affected by severe life-threatening disorders of likely genetic origin undergoing WGS, and/or WES diagnostic test, either in an emergency setting (i.e., neonatal intensive care unit or NICU and paediatric intensive care unit or PICU) or in an outpatient setting. Where available in the included studies, UC was also considered. UC (e.g., chromosomal micro-array [CMA], targeted gene panel, array CGH, fluorescence in-situ hybridization, karyotype) was defined as sequencing methods not involving massively parallel sequencing and not allowing to screen simultaneously for mutations in hundreds of loci in genetically heterogeneous disorders, whole-genome screening for novel mutations, and sequence-based detection of novel pathogens that cause human diseases [29]. The inclusion was restricted to articles written in English and published between January 2010 and June 2022. The indicated timespan reflected the new sequencing technologies not available in older publications or being outdated owing to technological developments [30]. The search strategy was further restricted by availability of full texts published in peer-reviewed journals and by type of articles, which excluded non-primary literature, as commentary, books, thesis, and reviews. Assessment of the eligibility criteria was carried out independently by three authors; in the case of divergence, a fourth author was consulted. The primary outcome of our search was the diagnostic yield which was measured as the number of patients in which the genetic test suggested the definitive diagnosis out of the total number of patients undergoing the test. After the removal of duplicate articles, and according to the inclusion and exclusion criteria, three independent researchers performed the preliminary screening by evaluating the titles and abstracts. Then, the same subjects screened the full text of each study to determine the potential eligibility. In both screening phases, all disagreements were solved by a fourth author by discussing the inclusion and exclusion criteria of the article.

### Data analysis

Data extraction was completed by three independent investigators. A pre-determined data extraction spreadsheet was designed including the following variables: study characteristics, country of the study, setting, sample size, age, intervention, comparator, indicators, and main findings. Methodological quality of studies evaluating diagnostic yield was assessed using the Quality Assessment of Diagnostic Accuracy studies (QUADAS-2) scale [29] as recommended by the Agency for Healthcare Research and Quality (AHRQ), the Cochrane Collaboration, and the National Institute for Health and Clinical Excellence (NICE). The use of QUADAS-2 implies four phases: (1) state the review question, (2) develop review specific guidance, (3) review the published flow diagram for the primary study or construction of a flow diagram if none is reported, and (4) judgement of bias and applicability. The scale includes four domains: (1) patient selection, (2) index test, (3) reference standard, and (4) flow and timing. The first part of each key domain regards bias and includes information used to support the risk of bias judgment, a set of signalling questions to help reviewers reach the judgements regarding bias, and judgment of risk of bias. For each signalling question, the investigator could select “yes,” “no,” or “unclear”. The risk of bias was judged as “low”, “high” or “unclear”. If all signalling questions for each domain answered “yes”, the risk of bias was judged “low”, while, if any signalling question answered “no”, the risk of bias was considered “high”. The term “unclear” was used whenever the risk of bias could not be assessed due to missing information. The second part of the first three domains regarded applicability. The applicability sections were similar to the bias sections except for the signalling questions. Concerns regarding applicability were rated as “low”, “high” or “unclear”, the latter definition being used when insufficient data were reported. Studies rated as “low” on all domains regarding bias or applicability received an overall judgment of “low risk of bias” or “low concern regarding applicability”. Studies rated as “high” in one or more domains were judged “at risk of bias” or as having “concern regarding applicability”. QUADAS-2 assessments were summarized through the relative tabular and graphical displays [30].

The Grading of Recommendations, Assessment, Development and Evaluations (GRADE) approach was adopted to assess the evidence quality for the outcome of interest across the included studies. The GRADE method categorizes the level of evidence quality into: high quality, further research is extremely unlikely to change the credibility of the pooled results; moderate quality, further research is likely to influence the credibility of pooled results and may change the estimate; low quality, further research is extremely likely to influence the credibility of pooled results and is likely to change the estimate; very low quality, the pooled results have extreme uncertainty [31]. The online GRADE profiler (GRADEpro) [32] was adopted to create evidence profiles and summary of findings tables. For the outcome of interest, the quality of evidence was downgraded if the risk of bias, inconsistency, indirectness, imprecision, and publication bias were assessed as having serious limitations [33]. The overall quality was rated as either “high”, “moderate”, “low”, or “very low”.

Quantitative data synthesis was performed by always keeping the diagnostic yield, in terms of proportion of cases detected out of the total, as outcome of reference. Firstly, the diagnostic yield was meta-analysed, by inspecting differences between diverse techniques (WES, WGS, and UC) via subgroup analyses: to this purpose, in view of the expected heterogeneity among studies [34], random-effects models were developed according to DerSimonian and Laird [35] and heterogeneity was inspected using the I^2^ statistic (threshold level for significant heterogeneity: ≥ 50%) and chi-squared test for homogeneity (significance level for heterogeneity: *p* < 0.1) [36]. Given the available number of studies, a meta-regression model was also built, in order to compare the techniques by adjusting for relevant covariates (ICU *vs* non-ICU setting, Mendelian *vs* non-Mendelian disease, publication before *vs* after 2017). Another meta-regression model was run stratifying by the value (i.e., low and high) of the diagnostic yield reported by the primary studies included in the revision. The cut-off value was set according to the pooled diagnostic yield estimated through the random-effects meta-analysis.

Secondly, a network meta-analysis was performed by considering all studies comparing at least two of the three techniques. A frequentist approach based on the Mantel–Haenszel method for binary data, as described by Efthimiou et al. [37], was adopted. Heterogeneity was quantified through the test of inconsistency (Cochran’s Q statistic), and the odds ratio was chosen as summary measure, as widely recommended for indirect comparisons of binary variables because of the symmetry and invariance of this measure to the coding of event and non-event [38–40].

All statistical analyses were carried out, and plots were drawn, using the statistical software R (version 4.0.5) [41]: specifically, the “meta” package (version 5.0.0) [42] was used for the meta-analysis of proportions and meta-regression, while the “netmeta” package (version 2.0–0) was used for the network meta-analysis [43]. Two-sided *p*-values < 0.05 were considered statistically significant.

### Role of the funding source

The funding source had no involvement in study design; in the collection, analysis and interpretation of data; in the writing of the report; and in the decision to submit the article for publication.

## Results

**Fig. 1:**
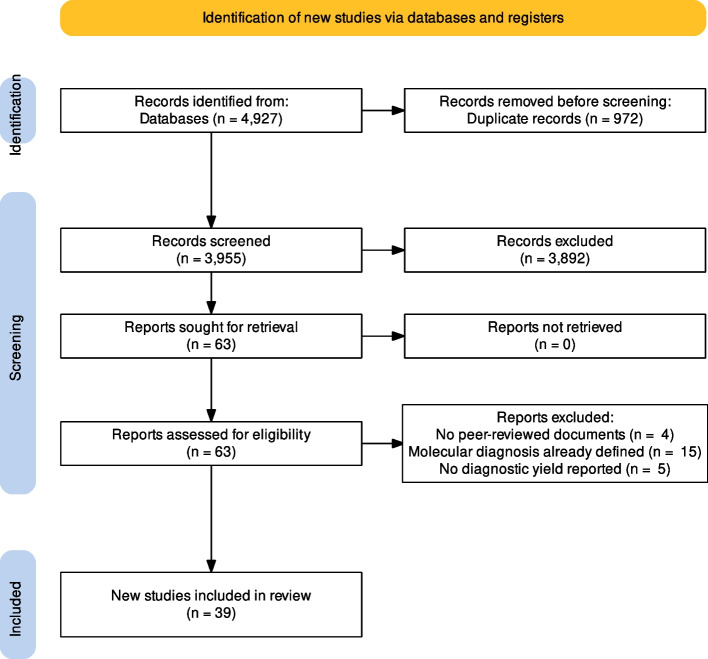
PRISMA flow diagram related to the included studies in the meta- analysis

The database search resulted in 4,927 publications and 18 studies were retrieved through the snowball search method. After duplicates elimination, 3,955 titles and abstracts were screened. A total of 63 articles were identified for a full-text screening. After full-text examination, 24 papers were excluded since they did not fulfil the eligibility criteria. Thus, 39 [15, 16, 24, 44–79] articles were included in the systematic review and were also considered for the meta-analysis (Fig. 1).

The considered manuscripts were published between 2015 and 2022, including 17 [15, 24, 44, 47, 49, 56, 58, 61–64, 66, 67, 70, 76, 78, 79] in USA, 7 in China [45, 46, 57, 65, 69, 74, 75], 2 in Canada [60, 80], 2 in Australia [52, 54], 2 in the UK [51, 71], and 1 in France [50], Poland [16], the Netherlands [48], Germany [72], Turkey [77], Saudi Arabia [59], Malaysia [73], Mexico [55], and Brazil [68]. Twenty-two papers were retrospective cohort studies [15, 24, 45, 47, 49–51, 55, 56, 63, 64, 66–68, 70, 71, 73, 75, 77–79], fourteen were prospective cohort studies [16, 44, 46, 48, 52–54, 57, 59, 60, 65, 72, 74], and three were randomized controlled trials (RCT) [58, 61, 76]. The mean age of enrolled children varied from 2 days to less than 18 years.

All the included articles estimated the diagnostic yield, 12 [15, 24, 47, 49, 52, 54, 55, 57, 67, 69, 75, 76] considered also the change in clinical management, four studies estimated the healthcare resource utilization [48, 49, 63, 79], and only one study [47] also assessed the 120-day mortality. Table S1, in the supplementary file, provides a summary of the main characteristics of each of the 39 publications.

**Fig. 2:**
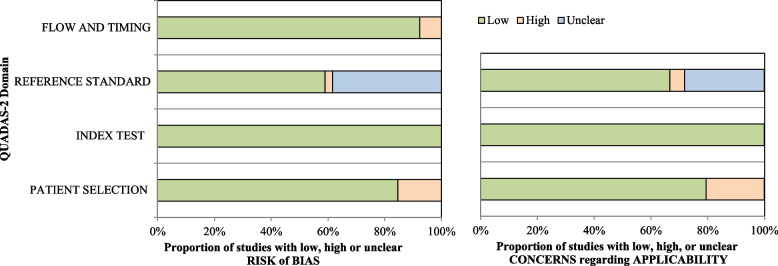
Stacked bar charts of Quality Assessment of Diagnostic Accuracy Studies -2 (QUADAS-2) scores showing an overview of the methodological quality of included studies, expressed as a percentage of studies that met each criterion

The overall methodological quality within individual studies is summarized in Table S2 of supplementary file and Fig. 2. Almost half of the studies were deemed at low risk for bias in all the domains [16, 44, 46–48, 50, 52–55, 58, 60, 62, 65, 69, 72, 76, 77]. As for the third domain, reference standard, one studies [78] resulted in high risk of bias because information about blind assessment were not reported. In the fourth domain, flow and timing, three studies [59, 63, 68] had high risk of bias, as not all patients received the same reference standard and were not included in the final analysis.

Nine studies [24, 56, 64, 66–68, 70, 71, 73] got a high risk of bias in the applicability section, particularly in the patient selection, as there may be issues related to the enrolment of patients. Eleven studies [49, 51, 56, 57, 61, 64, 66, 67, 74, 75, 79] got an unclear risk of bias in the applicability section, more specifically in the reference standard as it was not clear if its interpretation could have influenced the diagnostic accuracy estimates.

The results of assessment of quality evidence are shown in the supplementary material (i.e., Table S3 and Table S4). Overall, the quality of evidence from the outcome evaluated was recommended by the GRADE system as moderate for the thirty-six observational studies [15, 16, 24, 44–57, 59, 60, 62–75, 77–79] and as high for the three RCTs [58, 61, 76].

**Fig. 3:**
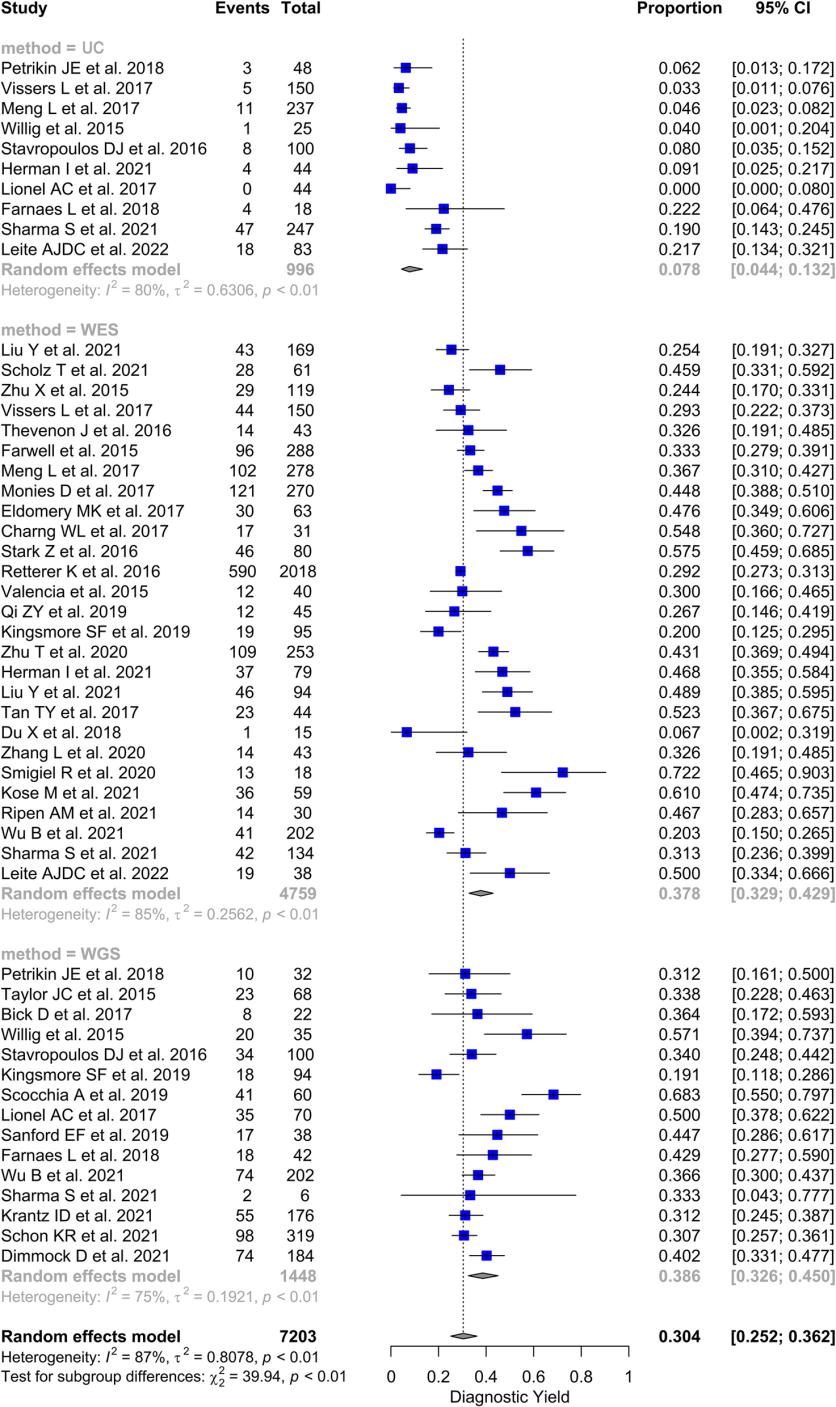
Forest plot of the diagnostic yield of usual care, WES and WGS, reported in the studies included in the systematic review and meta-analysis, 2015–2022

All the included studies evaluated the diagnostic yield of at least one technique among WGS and WES. and UC. Diagnostic yield proportions ranged from 19.1 to 68.3% for WGS (15 studies) [24, 47, 51, 53, 55, 58, 60, 61, 63, 67, 69–71, 76, 79], from 6.7 to 72.2% for WES (27 studies) [15, 16, 44–46, 48–50, 52, 54, 56, 57, 59, 61, 62, 64–66, 68–70, 72–75, 77, 78], and from 0 to 22.2% for UC (10 studies) [15, 47, 48, 53, 58, 60, 63, 68, 70, 78]. Meta-analytic synthesis yielded pooled diagnostic yield estimates of 7.8% (95% CI: [4.4 – 13.2]) for UC, 37.8% (95% CI: [32.9 – 42.9]) for WES and 38.6% (95% CI: [32.6 – 45.0]) for WGS (Fig. 3).

The meta-regression output suggested a possible trend towards a greater diagnostic yield of the WGS technique compared to WES after controlling for relevant covariates, although not attaining statistical significance (adjusted OR: 1.13 [95% CI: 0.79 – 1.62], *p *= 0.5001). Full detail of the meta-regression coefficients is reported in Table 1: of note, the confounding effect was particularly evident for the type of disease (monogenic vs non-monogenic, *p* = 0.0174) and – to a lesser extent – for the setting (ICU vs non-ICU, *p* = 0.1317), with a tendency to a better diagnostic performance for Mendelian diseases and in non-ICU settings respectively. Stratifying by the value of diagnostic yield (i.e., low and high), the effect for the type of disease is more evident in studies reporting a diagnostic yield higher than the pooled value (Table S5 and Table S6).

**Table 1:** Meta-regression analysis

	**Adjusted Beta**	**Odds Ratio [95% CI]**	***p*-value**
Technique (reference = *WES*)			
* UC*	-1.763	0.17 [0.11—0.27]	< 0.0001
* WGS*	0.124	1.13 [0.79—1.62]	0.5001
Monogenic disease, *yes*	0.399	1.49 [1.07—2.07]	0.0174
NICU/PICU setting, *yes*	-0.252	0.78 [0.56—1.08]	0.1317
Publication date, *after 2017* (reference = *before 2017*)	0.178	1.19 [0.83—1.73]	0.5452

*Abbreviations*: *CI* Confidence interval, *WES*, Whole exome sequencing, *WGS* Whole genome sequencing, *NICU* Neonatal intensive care unit, *PICU* Paediatric intensive care unit, *UC* Usual care

**Fig. 4:**
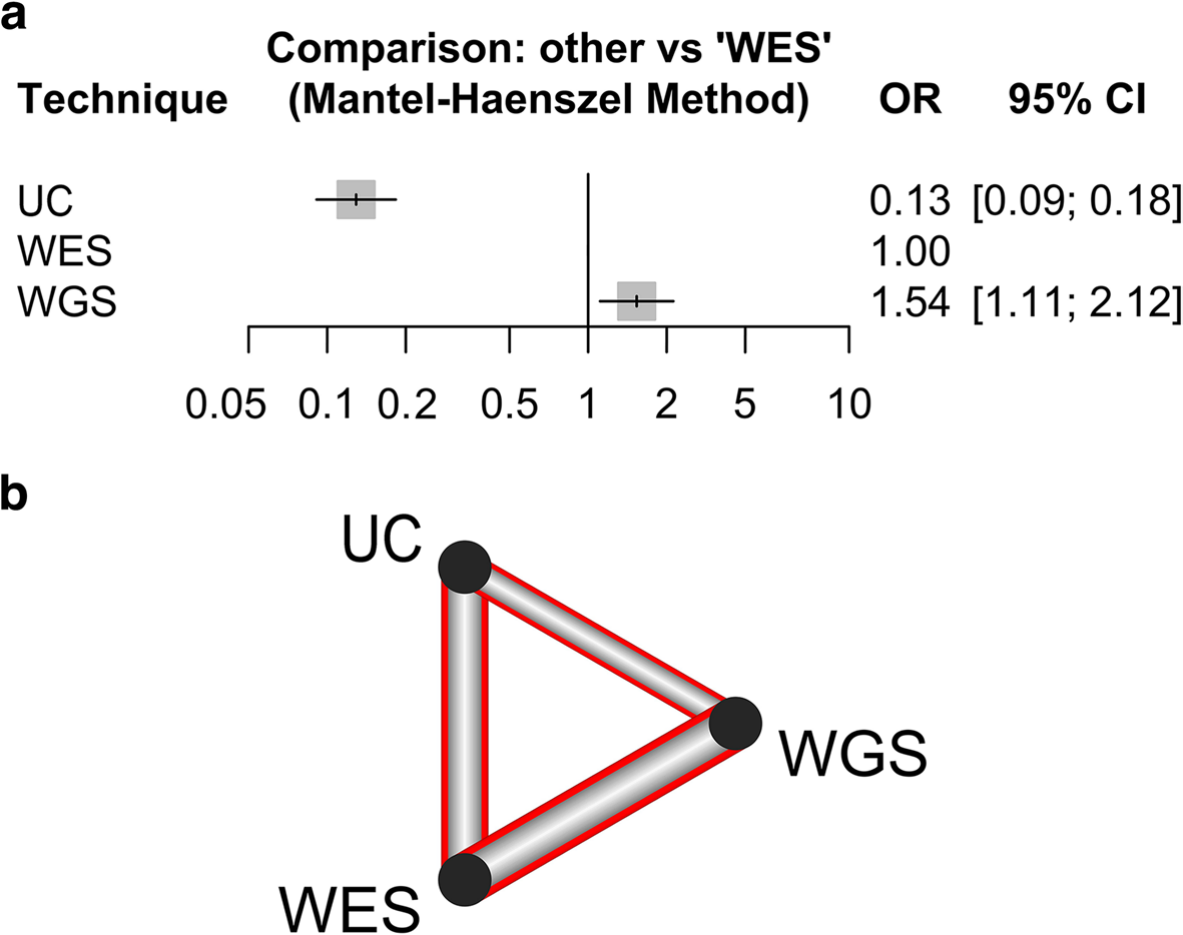
Results of the network meta-analysis comparing different diagnostic techniques. **a** Forest plot. Estimates are reported in the form of odds ratios, and the WES test is taken as reference. **b** Diagnostic yield network diagram. Red highlighting means significant difference between techniques (i.e., all relationships are significant). Thickness is proportional to the inverse standard error of each model comparing two techniques

Furthermore, twelve studies comparing two of the three techniques were included in the network meta-analysis: four studies compared UC against WES, five UC against WGS and only three directly compared WES against WGS (one of these also showed results for usual care). Besides confirming the superior performances of sequencing techniques over usual care, the network forest plot suggests a higher diagnostic yield for WGS compared to WES (OR = 1.54, 95%CI: [1.11 – 2.12], Fig. 4a).

As depicted by the network diagram (Fig. 4b), all pairwise comparisons between techniques showed statistically significant differences.

## Discussion

The present study suggests a higher diagnostic yield of WGS, with respect to WES (OR = 1.54, 95%CI: [1.11 – 2.12]) and UC, for paediatric patients with suspected genetic disorders, with a propensity to better diagnostic performances for Mendelian diseases.

The combination of study findings provides support for a main implication that, despite an overall difference, in terms of diagnostic yield, of 2% between WES and WGS, the latter is notably suitable for a specific subgroup of patients (i.e., paediatric patients with suspected Mendelian disorders) in whom the diagnostic yield is 50% higher with respect to patients with suspected non-monogenic diseases.

Therefore, the adoption of WGS should be taken considering the different priorities characterizing at different level (i.e., macro-, meso-, and micro-level) of the decision-making process in healthcare system. At macro-level, policymakers should assess the sustainability of this technology, consistently recognize the mechanisms underlying its overall financing, and try to define tailored diagnosis-related groups (DRG) tariffs for the reimbursement of the inpatient health services specific to this innovative diagnostic test. At meso-level, healthcare providers should oversee the acquisition and monitoring of WGS use. At micro-level, healthcare professionals should develop the competencies for its provision across different health settings. In a perspective of healthcare sustainability, it is crucial to develop sound genomic policies and programs that take into account WGS by implementing the three core functions (i.e., assessment, policy development, and assurance) to the provision of this genetic technique in health-care services [81].

[86]Another notable implication of whole genome sequencing (WGS) is that its wider utilization in diagnosis, which entails earlier and more accurate disease management, may limit the individual and societal impacts of disease, such as reducing the need for expensive and invasive follow-up testing or procedures and minimizing disease-related disability or mortality. This, in turn, could prevent or mitigate future burdens on healthcare systems in terms of both costs and outcomes [83].Nevertheless, nowadays, it is noteworthy to highlight the large availability of WES in respect of the still limited adoption of WGS in the clinical practice. Therefore, being cognizant of a significant difference between WGS and WES in terms of costs and complexity in interpreting data as well as the still slight gain in diagnostic yield of WGS over WES, there could be delays and hurdles in transferring WGS into the routine clinical workup.

The present systematic review and meta-analysis should be considered in light of its main strengths and limitations. First of all, the accurate literature review, the detailed quality assessment, the meticulous GRADE assessment of evidence quality, and the accurate meta-analysis are strengths of this study. Furthermore, the quite elevated number of retrieved studies and the different geographical areas, in which they are conducted, could increase the generalizability of the present systematic review.

The majority of (92%) studies considered for GRADE assessment adopted an observational study design, even though the rigorous methodology for assessing the evidence quality across the studies was followed. The papers included for pooled analyses reported a considerable heterogeneity, albeit it can be justified by the clinical and methodological diversity (i.e., different sample size). Another caveat of this article is that subgroup analysis on the diagnostic yield of specific investigated conditions, in the included papers, was not conducted, which may limit the generalizability of the findings to certain diseases. Moreover, it was not possible to fully investigate the genetic underpinnings of the investigated conditions due to the lack of available primary data on the specific sequenced mutations, thus limiting the superiority of WGS over the other sequencing techniques.

Over the last few years, the cost of WGS has drop down markedly potentially bringing it within the realm of cost-effectiveness for high-intensity medical practice, such as occurs in NICUs [17]. WGS has several advantages over other sequencing methods. Firstly, it offers the possibility to perform Mendelian gene discovery, which involves identifying the genetic basis of rare inherited disorders. Additionally, it has the potential to identify modifier genes, which are genes that modify the severity or course of a disease caused by a primary genetic mutation. Another advantage of WGS is that a single genome-wide test can replace multiple panel tests, saving time and resources and shortening the "diagnostic odysseys". WGS also allows the creation of sufficiently large genome-wide datasets, which might be used to predict the risk of developing further complex diseases. Finally, WGS can sequence non-coding variants and detect large insertions/deletions usually undetected by WES and UCT, providing a more comprehensive picture of an individual’s genome [82]. Further research rigorously assessing costs, effectiveness, cost-effectiveness, organizational impacts, ethical aspects of WGS in a health technology assessment perspective [83] in a transparent manner is mandatory to allow for a more informed decision-making process in this context.

Moreover, additional primary studies (preferably high-quality RCTs with larger samples) firstly evaluating the comparison between WGS and WES and then between WGS and standard genetic testing, are required to deeply investigate the differences in costs and diagnostic yield and to increase the level of quality evidence.

## Conclusion

Whole genome sequencing for the paediatric population with suspected genetic disorders allows an accurate and early genetic diagnosis in a high proportion of cases. This provides understanding of the molecular mechanism underlying diseases, supports tailored treatments and accurate genetic counselling, while reduces the burden of unsolved cases that weigh on patients and their families [25] by putting end to the so called “diagnostic Odyssey” [84, 85]. The present review suggests the use of WGS in the diagnostic workup of ill paediatric patients with suspected genetic disorders strengthened by evidence at policy, program, and intervention levels. Our study also reinforces the use of methodologies capable of providing robust evidence for the formulation of health policy on funding, to overcome present hurdles in transitioning WGS from the research setting into routine clinical practice. However, there is a pressing issue of efficiently allocating limited healthcare resources for HTA agencies when it comes to WGS approaches. Overcoming this challenge will be critical to realizing the potential benefits of WGS for improving patient outcomes and reducing healthcare costs.

## Supplementary Information

**Additional file 1.** Aims and scope.

**Additional file 2: Table S1.** Characteristics of the studies included in the systematic review andmeta-analysis, 2015–2022. **Table S2.** Results of the Quality Assessment of Diagnostic Accuracy Studies -2 (QUADAS-2) tool for studiesincluded in systematic review and meta-analysis, 2015–2022. **Table S3.** GRADE Evidence Profiles for studies included in the systematic review and meta-analysis, 2015–2022. **Table S4.** Summary of findings table for studies included in the systematic review and meta-analysis, 2015–2022. **Table S5.** Meta-regression analysis considering studies with lower diagnostic yield (i.e., inferior to the pooledestimate for each diagnostic technique). **Table S6.** Meta-regression analysis considering studies with higher diagnostic yield (i.e., superior to the pooled estimate for each diagnostic technique).

## Data Availability

Aggregated data and materials might be made available upon reasonable request via email to the corresponding author.

## References

[1] Amberger JS, Bocchini CA, Schiettecatte F, Scott AF, Hamosh A (2015). OMIM.org: Online Mendelian Inheritance in Man (OMIM®), an online catalog of human genes and genetic disorders. Nucleic Acids Res.

[2] McKusick-Nathans Institute of Genetic Medicine JHU (2021). Online Mendelian Inheritance in Man, OMIM®.

[3] Online Mendelian Inheritance in Man® (2021). OMIM Entry Statistics.

[4] Orphanet (2021). Orphanet Reports Series / Procedures.

[5] Wright CF, FitzPatrick DR, Firth HV (2018). Paediatric genomics: diagnosing rare disease in children. Nat Rev Genet.

[6] Rahit KMTH, Tarailo-Graovac M (2020). Genetic Modifiers and Rare Mendelian Disease. Genes (Basel).

[7] EURORDIS (2020). About Rare Diseases.

[8] Richter T, Nestler-Parr S, Babela R, Khan ZM, Tesoro T, Molsen E (2015). Rare Disease Terminology and Definitions—A Systematic Global Review: Report of the ISPOR Rare Disease Special Interest Group. Value Health.

[9] Nguengang Wakap S, Lambert DM, Olry A, Rodwell C, Gueydan C, Lanneau V (2020). Estimating cumulative point prevalence of rare diseases: analysis of the Orphanet database. Eur J Hum Genet.

[10] Kole, Anna, Faurisson François, Mavris M (2009). The Voice of 12,000 Patients.

[11] Lu JT, Campeau PM, Lee BH (2014). Genotype-Phenotype Correlation — Promiscuity in the Era of Next-Generation Sequencing. N Engl J Med.

[12] Radio FC, Ruzzeddu M, Bartuli A, Novelli A, Tartaglia M, Dallapiccola B. Cost-effectiveness of exome sequencing: an Italian pilot study on undiagnosed patients. New Genet Soc 2019;38. 10.1080/14636778.2019.1601008.

[13] Rae W, Ward D, Mattocks C, Pengelly RJ, Eren E, Patel SV (2018). Clinical efficacy of a next-generation sequencing gene panel for primary immunodeficiency diagnostics. Clin Genet.

[14] French CE, Delon I, Dolling H, Sanchis-Juan A, Shamardina O, Mégy K (2019). Whole genome sequencing reveals that genetic conditions are frequent in intensively ill children. Intensive Care Med.

[15] Meng L, Pammi M, Saronwala A, Magoulas P, Ghazi AR, Vetrini F (2017). Use of Exome Sequencing for Infants in Intensive Care Units. JAMA Pediatr.

[16] Śmigiel R, Biela M, Szmyd K, Błoch M, Szmida E, Skiba P (2020). Rapid Whole-Exome Sequencing as a Diagnostic Tool in a Neonatal/Pediatric Intensive Care Unit. J Clin Med.

[17] Saunders CJ, Miller NA, Soden SE, Dinwiddie DL, Noll A, Alnadi NA (2012). Rapid Whole-Genome Sequencing for Genetic Disease Diagnosis in Neonatal Intensive Care Units. Sci Transl Med.

[18] Barbitoff YA, Polev DE, Glotov AS, Serebryakova EA, Shcherbakova IV, Kiselev AM (2020). Systematic dissection of biases in whole-exome and whole-genome sequencing reveals major determinants of coding sequence coverage. Sci Rep.

[19] van Nimwegen KJM, van Soest RA, Veltman JA, Nelen MR, van der Wilt GJ, Vissers LELM (2016). Is the $1000 Genome as Near as We Think? A Cost Analysis of Next-Generation Sequencing. Clin Chem.

[20] Radio FC, Ruzzeddu M, Bartuli A, Novelli A, Tartaglia M, Dallapiccola B (2019). Cost-effectiveness of exome sequencing: an Italian pilot study on undiagnosed patients. New Genet Soc.

[21] Mattick JS, Dinger M, Schonrock N, Cowley M (2018). Whole genome sequencing provides better diagnostic yield and future value than whole exome sequencing. Med J Aust.

[22] Clark MM, Stark Z, Farnaes L, Tan TY, White SM, Dimmock D (2018). Meta-analysis of the diagnostic and clinical utility of genome and exome sequencing and chromosomal microarray in children with suspected genetic diseases. NPJ Genom Med.

[23] Schwarze K, Buchanan J, Taylor JC, Wordsworth S (2018). Are whole-exome and whole-genome sequencing approaches cost-effective? A systematic review of the literature. Genet Med.

[24] Sanford EF, Clark MM, Farnaes L, Williams MR, Perry JC, Ingulli EG (2019). Rapid Whole Genome Sequencing Has Clinical Utility in Children in the PICU*. Pediatr Crit Care Med.

[25] Marshall CR, Bick D, Belmont JW, Taylor SL, Ashley E, Dimmock D (2020). The Medical Genome Initiative: moving whole-genome sequencing for rare disease diagnosis to the clinic. Genome Med.

[26] Daoud H, Luco SM, Li R, Bareke E, Beaulieu C, Jarinova O (2016). Next-generation sequencing for diagnosis of rare diseases in the neonatal intensive care unit. Can Med Assoc J.

[27] O’Connor, Denise Ann. Green, Sally Elizabeth. Higgins JP. Defining the review question and developing criteria for including studies. In: Green, Sally Elizabeth. Higgins JP, editor. Cochrane Handbook for Systematic Reviews of Interventions. First: John Wiley & Sons, Ltd; 2008, 83–94.

[28] Moher D, Liberati A, Tetzlaff J, Altman DG (2009). Preferred reporting items for systematic reviews and meta-analyses: the PRISMA statement. BMJ.

[29] Whiting PF (2011). QUADAS-2: A Revised Tool for the Quality Assessment of Diagnostic Accuracy Studies. Ann Intern Med.

[30] University of Bristol (2020). QUADAS-2: Background Document.

[31] Guyatt G, Oxman AD, Akl EA, Kunz R, Vist G, Brozek J (2011). GRADE guidelines: 1. Introduction—GRADE evidence profiles and summary of findings tables. J Clin Epidemiol.

[32] McMaster University and Evidence Prime Inc (2020). GRADEpro GDT: GRADEpro Guideline Development Tool [Software].

[33] Balshem H, Helfand M, Schünemann HJ, Oxman AD, Kunz R, Brozek J (2011). GRADE guidelines: 3. Rating the quality of evidence. J Clin Epidemiol.

[34] Borenstein M, Hedges L v, Higgins JPT, Rothstein HR (2010). A basic introduction to fixed-effect and random-effects models for meta-analysis. Res Synth Methods.

[35] DerSimonian R, Laird N (2015). Meta-analysis in clinical trials revisited. Contemp Clin Trials.

[36] Higgins JPT (2003). Measuring inconsistency in meta-analyses. BMJ.

[37] Efthimiou O, Rücker G, Schwarzer G, Higgins JPT, Egger M, Salanti G (2019). Network meta-analysis of rare events using the Mantel-Haenszel method. Stat Med.

[38] Caldwell DM, Welton NJ, Dias S, Ades A (2012). Selecting the best scale for measuring treatment effect in a network meta-analysis: a case study in childhood nocturnal enuresis. Res Synth Methods.

[39] Coory M, Jordan S (2010). Frequency of treatment-effect modification affecting indirect comparisons. Pharmacoeconomics.

[40] Eckermann S, Coory M, Willan AR (2009). Indirect comparison: relative risk fallacies and odds solution. J Clin Epidemiol.

[41] R Core Team. R: A language and environment for statistical computing. R Foundation for Statistical Computing 2021. https://www.r-project.org/ (Accessed 10 Feb 2022).

[42] Balduzzi S, Rücker G, Schwarzer G (2019). How to perform a meta-analysis with R: a practical tutorial. Evid Based Mental Health.

[43] Rücker G, Krahn U, König J, Efthimiou O, Davies A, Papakonstantinou T (2022). Package ‘netmeta'.

[44] Zhu X, Petrovski S, Xie P, Ruzzo EK, Lu Y-F, McSweeney KM (2015). Whole-exome sequencing in undiagnosed genetic diseases: interpreting 119 trios. Genet Med.

[45] Zhu T, Gong X, Bei F, Ma L, Chen Y, Zhang Y, et al. Application of Next-Generation Sequencing for Genetic Diagnosis in Neonatal Intensive Care Units: Results of a Multicenter Study in China. Front Genet 2020;11. 10.3389/fgene.2020.565078. 10.3389/fgene.2020.565078 PMC7677510 33240318

[46] Zhang L, Gao J, Liu H, Tian Y, Zhang X, Lei W (2020). Pathogenic variants identified by whole-exome sequencing in 43 patients with epilepsy. Hum Genomics.

[47] Willig LK, Petrikin JE, Smith LD, Saunders CJ, Thiffault I, Miller NA (2015). Whole-genome sequencing for identification of Mendelian disorders in critically ill infants: a retrospective analysis of diagnostic and clinical findings. Lancet Respir Med.

[48] Vissers LELM, van Nimwegen KJM, Schieving JH, Kamsteeg E-J, Kleefstra T, Yntema HG (2017). A clinical utility study of exome sequencing versus conventional genetic testing in pediatric neurology. Genet Med.

[49] Valencia CA, Husami A, Holle J, Johnson JA, Qian Y, Mathur A, et al. Clinical impact and cost-effectiveness of whole exome sequencing as a diagnostic tool: a pediatric center’s experience. Front Pediatr 2015;3. 10.3389/fped.2015.00067. 10.3389/fped.2015.00067 PMC4522872 26284228

[50] Thevenon J, Duffourd Y, Masurel-Paulet A, Lefebvre M, Feillet F, el Chehadeh-Djebbar S (2016). Diagnostic odyssey in severe neurodevelopmental disorders: toward clinical whole-exome sequencing as a first-line diagnostic test. Clin Genet.

[51] Taylor JC, Martin HC, Lise S, Broxholme J, Cazier J-B, Rimmer A (2015). Factors influencing success of clinical genome sequencing across a broad spectrum of disorders. Nat Genet.

[52] Tan TY, Dillon OJ, Stark Z, Schofield D, Alam K, Shrestha R (2017). Diagnostic Impact and Cost-effectiveness of Whole-Exome Sequencing for Ambulant Children With Suspected Monogenic Conditions. JAMA Pediatr.

[53] Stavropoulos DJ, Merico D, Jobling R, Bowdin S, Monfared N, Thiruvahindrapuram B (2016). Whole-genome sequencing expands diagnostic utility and improves clinical management in paediatric medicine. NPJ Genom Med.

[54] Stark Z, Tan TY, Chong B, Brett GR, Yap P, Walsh M (2016). A prospective evaluation of whole-exome sequencing as a first-tier molecular test in infants with suspected monogenic disorders. Genet Med.

[55] Scocchia A, Wigby KM, Masser-Frye D, del Campo M, Galarreta CI, Thorpe E (2019). Clinical whole genome sequencing as a first-tier test at a resource-limited dysmorphology clinic in Mexico. NPJ Genom Med.

[56] Retterer K, Juusola J, Cho MT, Vitazka P, Millan F, Gibellini F (2016). Clinical application of whole-exome sequencing across clinical indications. Genet Med.

[57] Qi ZY, Duan J, He XY, Zhong Q-H, Zhang CY, Xie YB (2019). [Clinical application of whole exome sequencing in monogenic hereditary disorders in critically ill newborns]. Zhongguo Dang Dai Er Ke Za Zhi.

[58] Petrikin JE, Cakici JA, Clark MM, Willig LK, Sweeney NM, Farrow EG (2018). The NSIGHT1-randomized controlled trial: rapid whole-genome sequencing for accelerated etiologic diagnosis in critically ill infants. NPJ Genom Med.

[59] Monies D, Abouelhoda M, AlSayed M, Alhassnan Z, Alotaibi M, Kayyali H (2017). The landscape of genetic diseases in Saudi Arabia based on the first 1000 diagnostic panels and exomes. Hum Genet.

[60] Lionel AC, Costain G, Monfared N, Walker S, Reuter MS, Hosseini SM (2018). Improved diagnostic yield compared with targeted gene sequencing panels suggests a role for whole-genome sequencing as a first-tier genetic test. Genet Med.

[61] Kingsmore SF, Cakici JA, Clark MM, Gaughran M, Feddock M, Batalov S (2019). A Randomized, Controlled Trial of the Analytic and Diagnostic Performance of Singleton and Trio, Rapid Genome and Exome Sequencing in Ill Infants. Am J HumGenet.

[62] Farwell KD, Shahmirzadi L, El-Khechen D, Powis Z, Chao EC, Tippin Davis B (2015). Enhanced utility of family-centered diagnostic exome sequencing with inheritance model–based analysis: results from 500 unselected families with undiagnosed genetic conditions. Genet Med.

[63] Farnaes L, Hildreth A, Sweeney NM, Clark MM, Chowdhury S, Nahas S (2018). Rapid whole-genome sequencing decreases infant morbidity and cost of hospitalization. NPJ Genom Med.

[64] Eldomery MK, Coban-Akdemir Z, Harel T, Rosenfeld JA, Gambin T, Stray-Pedersen A (2017). Lessons learned from additional research analyses of unsolved clinical exome cases. Genome Med.

[65] Du X, Gao X, Liu X, Shen L, Wang K, Fan Y, et al. Genetic Diagnostic Evaluation of Trio-Based Whole Exome Sequencing Among Children With Diagnosed or Suspected Autism Spectrum Disorder. Front Genet 2018;9. 10.3389/fgene.2018.00594. 10.3389/fgene.2018.00594 PMC6284054 30555518

[66] Charng W-L, Karaca E, Coban Akdemir Z, Gambin T, Atik MM, Gu S (2016). Exome sequencing in mostly consanguineous Arab families with neurologic disease provides a high potential molecular diagnosis rate. BMC Med Genomics.

[67] Bick D, Fraser P, Gutzeit M, Harris J, Hambuch T, Helbling D (2016). Successful Application of Whole Genome Sequencing in a Medical Genetics Clinic. J Pediatr Genet.

[68] da Leite AJC, Pinto IP, Leijsten N, Ruiterkamp-Versteeg M, Pfundt R, de Leeuw N (2022). Diagnostic yield of patients with undiagnosed intellectual disability, global developmental delay and multiples congenital anomalies using karyotype, microarray analysis, whole exome sequencing from Central Brazil. PLoS One.

[69] Wu B, Kang W, Wang Y, Zhuang D, Chen L, Li L (2021). Application of Full-Spectrum Rapid Clinical Genome Sequencing Improves Diagnostic Rate and Clinical Outcomes in Critically Ill Infants in the China Neonatal Genomes Project*. Crit Care Med.

[70] Sharma S, Repnikova E, Noel-MacDonnell JR, LePichon J (2021). Diagnostic yield of genetic testing in 324 infants with hypotonia. Clin Genet.

[71] Schon KR, Horvath R, Wei W, Calabrese C, Tucci A, Ibañez K, et al. Use of whole genome sequencing to determine genetic basis of suspected mitochondrial disorders: cohort study. BMJ 2021:e066288. 10.1136/bmj-2021-066288. 10.1136/bmj-2021-066288 PMC8565085 34732400

[72] Scholz T, Blohm ME, Kortüm F, Bierhals T, Lessel D, van der Ven AT (2021). Whole-exome sequencing in critically Ill neonates and infants: diagnostic yield and predictability of monogenic diagnosis. Neonatology.

[73] Ripen AM, Chear CT, Baharin MF, Nallusamy R, Chan KC, Kassim A (2021). A single-center pilot study in Malaysia on the clinical utility of whole-exome sequencing for inborn errors of immunity. Clin Exp Immunol.

[74] Liu Y, Liu X, Qin D, Zhao Y, Cao X, Deng X (2021). Clinical utility of next-generation sequencing for developmental disorders in the rehabilitation department: experiences from a Single Chinese Center. J Mol Neurosci.

[75] Liu Y, Hao C, Li K, Hu X, Gao H, Zeng J, et al. Clinical application of whole exome sequencing for monogenic disorders in PICU of China. Front Genet 2021;12. 10.3389/fgene.2021.677699. 10.3389/fgene.2021.677699 PMC8440967 34539730

[76] Krantz ID, Medne L, Weatherly JM, Wild KT, Biswas S, Devkota B (2021). Effect of Whole-Genome Sequencing on the Clinical Management of Acutely Ill Infants With Suspected Genetic Disease. JAMA Pediatr.

[77] Kose M, Isik E, Aykut A, Durmaz A, Kose E, Ersoy M (2021). The utility of next-generation sequencing technologies in diagnosis of Mendelian mitochondrial diseases and reflections on clinical spectrum. J Pediatr Endocrinol Metab.

[78] Herman I, Lopez MA, Marafi D, Pehlivan D, Calame DG, Abid F (2021). Clinical exome sequencing in the diagnosis of pediatric neuromuscular disease. Muscle Nerve.

[79] Dimmock D, Caylor S, Waldman B, Benson W, Ashburner C, Carmichael JL (2021). Project Baby Bear: Rapid precision care incorporating rWGS in 5 California children’s hospitals demonstrates improved clinical outcomes and reduced costs of care. Am J Hum Genet.

[80] Stavropoulos DJ, Merico D, Jobling R, Bowdin S, Monfared N, Thiruvahindrapuram B (2016). Whole-genome sequencing expands diagnostic utility and improves clinical management in paediatric medicine. NPJ Genom Med.

[81] Brand A, Brand H, Schulte in den Bäumen T (2008). The impact of genetics and genomics on public health. Eur J Hum Genet.

[82] Mazzarotto F, Olivotto I, Walsh R (2020). Advantages and perils of clinical whole-exome and whole-genome sequencing in cardiomyopathy. Cardiovasc Drugs Ther.

[83] EUnetHTA Joint Action 2 WP 8 (2016). HTA Core Model ® version 3.0.

[84] Abolhassani H, Aghamohammadi A, Fang M, Rezaei N, Jiang C, Liu X (2019). Clinical implications of systematic phenotyping and exome sequencing in patients with primary antibody deficiency. Genet Med.

[85] Berberich AJ, Ho R, Hegele RA (2018). Whole genome sequencing in the clinic: empowerment or too much information?. Can Med Assoc J.

