# Supplementary material for: Whole genome sequencing diagnostic yield for paediatric patients with suspected genetic disorders: systematic review, meta-analysis, and GRADE assessment
Source: Arch Public Health. 2023 May 25;81:93. doi: 10.1186/s13690-023-01112-4 (PMC10210272; doi:10.1186/s13690-023-01112-4)
Supplement: Supplementary file 1 — Additional file 1. Aims and scope. [file 13690_2023_1112_MOESM1_ESM.docx]

**Aims and scope**

**What is known**

During the last years, the technological revolution of the healthcare system brought to the development of next generation sequencing (NGS) technologies and bioinformatic pipelines to manage and analyse genomic data. This phenomenon along with an impressive reduction of sequencing costs, allowed a wider adoption of genomic sequencing, especially regarding whole exome sequencing (WES). These tools have the potentiality to early identify a large number of molecular defects underlying Mendelian disorders, allowing the possibility for genomic medicine to become an important preventive instrument, besides its diagnostic and therapeutic role, with great potential to improve outcomes and to reduce costs in primary care setting. The application of whole genome sequencing (WGS) and the whole exome sequencing (WES) in new-borns and children suffering from a severe disorder of likely genetic origin is expected to improve targeted, effective care and management.

WGS and WES are already increasingly used for diagnostic purposes on critically ill infants and children admitted to Neonatal Intensive Care Units (NICU) and Paediatric Intensive Care Units (PICU) with a suspected genetic disorder. Traditional genetic testing allows to reach the diagnosis in around 20% of cases. Thus, acutely ill neonates with suspected genetic diseases are often discharged or deceased before diagnosis. As a result, NICU treatment of genetic diseases is usually empirical, may lack efficacy, may be inappropriate, or even may cause adverse effects.

**What does the study adds**

The present study suggests a higher diagnostic yield of WGS, with respect to WES (OR = 1.54, 95%CI: [1.11 – 2.12]) and usual care, for paediatric patients with suspected genetic disorders, with a propensity to better diagnostic performances for Mendelian diseases.

The combination of study findings provides support for a main implication that, despite an overall difference, in terms of diagnostic yield, of 2% between WES and WGS, the latter is notably suitable for a specific subgroup of patients (i.e., paediatric patients with suspected Mendelian disorders) in whom the diagnostic yield is 50% higher with respect to patients with suspected non-monogenic diseases.

Another conceptual implication is that WGS, additional to be more widespread use in diagnosis, which involve early and more precise management, could limit the impact of disease both at the individual and societal level, preventing or limiting future burdensome consequences, in terms of healthcare costs and outcomes, on health systems.

**What the implications are for clinical practice, public health and/or research**.

Nevertheless, nowadays, it is noteworthy to highlight the large availability of WES in respect of the still limited adoption of WGS in the clinical practice. Therefore, being cognizant of a significant difference between WGS and WES in terms of costs and complexity in interpreting data as well as the still slight gain in diagnostic yield of WGS over WES, there could be delays and hurdles in transferring WGS into the routine clinical workup. Over the last few years, the cost of WGS has drop down markedly potentially bringing it within the realm of costeffectiveness for high-intensity medical practice, such as occurs in NICUs. Thus, further research rigorously assessing costs, effectiveness, cost-effectiveness, organizational impacts, ethical aspects of WGS in a health technology assessment perspective in a transparent manner is mandatory to allow for a more informed decisionmaking process in this context.

The resulting insights on the differences among different NGS techniques serve as a starting point to coordinate further progress on their application and will foster evidence-based collaboration. The collection of evidence in this field, arising from the increased number of cases, will be a fundamental tool for a fair identification of their application fields. This study itself might be considered an important decision tool for policy makers in the process of allocating funds for research and technological innovation based on scientific evidence.

2
