# Supplementary material for: Whole genome sequencing diagnostic yield for paediatric patients with suspected genetic disorders: systematic review, meta-analysis, and GRADE assessment
Source: Arch Public Health. 2023 May 25;81:93. doi: 10.1186/s13690-023-01112-4 (PMC10210272; doi:10.1186/s13690-023-01112-4)
Supplement: Supplementary file 2 — Additional file 2: Table S1. Characteristics of the studies included in the systematic review andmeta-analysis, 2015–2022. Table S2. Results of the Quality Assessment of Diagnostic Accuracy Studies -2 (QUADAS-2) tool for studiesincluded in systematic review and meta-analysis, 2015–2022. Table S3. GRADE Evidence Profiles for studies included in the systematic review and meta-analysis, 2015–2022. Table S4. Summary of findings table for studies included in the systematic review and meta-analysis, 2015–2022. Table S5. Meta-regression analysis considering studies with lower diagnostic yield (i.e., inferior to the pooledestimate for each diagnostic technique). Table S6. Meta-regression analysis considering studies with higher diagnostic yield (i.e., superior to the pooled estimate for each diagnostic technique). [file 13690_2023_1112_MOESM2_ESM.docx]

**Whole Genome Sequencing diagnostic yield for pediatric patients with suspected genetic disorders: Systematic review, meta-analysis, and GRADE assessment**

**Supplemental Material**

**Search string**

(“Newborn” OR “infant” OR “neonate” OR “child” OR “suckling” OR “pediatric population*” OR “paediatric population*”)

AND

(“whole genome sequencing”[MeSH Terms] OR ((“whole" AND "genome”) AND “sequencing”) OR “whole genome sequencing” OR WGS) OR (“whole exome sequencing”[MeSH Terms] OR ((“whole” AND “exome”) AND “sequencing”) OR “whole exome sequencing” OR “WES”)

AND

(“chromosomal micro array” OR CMA OR “array CGH” OR “micro array CGH” OR “CGH” OR “high-throughput nucleotide sequencing”[MeSH Terms] OR “high-throughput nucleotide sequencing” OR “next generation sequencing” OR “sanger sequencing” OR “usual care”)

AND

(“diagnostic yield” OR “diagnostic sensitivity” OR “clinical utility” OR “utility” OR “time to diagnosis” OR “survival rate” [MeSH Terms] OR “survival rate” OR “change in treatment” OR “quality-adjusted life years”[MeSH Terms] OR “quality-adjusted life years” OR “QALY” OR “effectiveness” OR “effect” OR “benefit” OR “consequence” OR “NICU” OR “PICU” OR “neonatal intensive care unit” OR “pediatric intensive care unit” OR “critical illness”[MeSH Terms] OR “critical illness”)

**Table S1.** Characteristics of the studies included in the systematic review and meta-analysis, 2015–2022.

| **Author, year, country** | **Reason for testing** | **Intervention** | **Setting** | **Comparator** | **Population** | **Age**  **(mean)** | **Indicators** | **Results** |
| --- | --- | --- | --- | --- | --- | --- | --- | --- |
| Farwell, 2015, USA | Ataxia,  Multiple congenital anomalies,  Epilepsy | WES | Outpatient | NA | 500 | 11 years | Diagnostic yield | A positive or likely positive result in a characterized gene was identified in 30% of patients (152/500). A novel gene finding was identified in 7.5% of patients (31/416). |
| Taylor JC, 2015, UK | Suspected monogenic disorders | WGS | Outpatient | NA | 156 | < 18 years | Diagnostic yield | Disease causing variants were identified in 21% of cases, rising to 34% (23/68) for Mendelian disorders and 57% (8/14) in trios. 32 potentially clinically actionable variants were discovered in 18 genes unrelated to the referral disorder, though only four were ultimately considered reportable. |
| Valencia CA, 2015, USA | Suspected unspecific genetic disorders | WES | Outpatient | Sanger sequencing | 40 | 7 years | Diagnostic yield  Change in clinical management  Health-care resource use | Of the first 40 clinical cases, genetic defects were identified in 12 (30%) patients, of which 47% of the mutations were previously unreported in the literature. Significantly, genetic diagnosis from WES led to altered patient medical management in positive cases. |
| Willig LK, 2015, USA | Suspected monogenic disorders | WGS | NICU/PICU | Standard care | 35 | 26 days | Diagnostic yield | 20 (57%) of 35 infants were diagnosed with a genetic disease by use of STATseq and three (9%) of 32 by use of standard genetic testing (p=0·0002). 13 (65%) of 20 STATseq diagnoses were associated with de-novo mutations. |
| Zhu X, 2015, USA | Suspected unspecific genetic disorders | WES | Outpatient | NA | 119 | 9 years | Diagnostic yield | A genetic diagnosis was obtained for 29 (24%) of patients. |
| Retterer K et al. 2016, USA | Suspected unspecific genetic disorders | WES | Outpatient | NA | 3040 | 11 years | Diagnostic yield | The overall diagnostic yield of WES was 28.8%. Of 2,091 cases in which secondary findings were analyzed, 6.2% (N = 129) had reportable pathogenic variants. In addition to cases with a definitive diagnosis, in 24.2% of cases a candidate gene was reported that may later be reclassified as being associated with a definitive diagnosis. |
| Stark Z, 2016, Australia | Suspected monogenic disorders | WES | Outpatient | Standard care | 80 | < 2 years | Diagnostic yield | Of 80 enrolled infants, 46 received a molecular genetic diagnosis through singleton WES (57.5%) compared with 11 (13.75%) who underwent standard investigations in the same patient group. Clinical management changed following exome diagnosis in 15 of 46 diagnosed participants (32.6%). |
| Stavropoulos DJ, 2016, Canada | Suspected unspecific genetic disorders | WGS | NICU/PICU | Chromosomal microarray | 100 | 5.5 years | Diagnostic yield | WGS identified genetic variants meeting clinical diagnostic criteria in 34% of cases, representing a fourfold increase in diagnostic rate over CMA (8%; P value = 1.42E − 05) alone and more than twofold increase in CMA plus targeted gene sequencing (13%; P value = 0.0009). WGS identified all rare clinically significant CNVs that were detected by CMA. |
| Thevenon J, 2016, France | Neurodevelopmental disorders | WES | Outpatient | NA | 43 | 14 years | Diagnostic yield  Change in clinical management | The diagnostic rate of WES reached 32.5% (14 out of 43 individuals).Genetic diagnosis had a direct impact on clinical management in 4 families, including a prenatal diagnostic test in one family. |
| Bick D, 2017, USA | Suspected monogenic disorders | WGS | Outpatient | Standard care | 22 | < 16 years | Diagnostic yield,  Change in clinical management | A 14% (3/22) diagnosis rate over 2 years was obtained; with subsequent reanalysis, this increased to 36% (8/22). In 75% (6/8) of diagnosed cases, the diagnosis affected treatment and/or medical surveillance. |
| Charng WL, 2017, USA | Suspected monogenic disorders | WES | Outpatient | Standard care | 31 | < 16 years | Diagnostic yield | Overall, a potential molecular diagnosis was provided by variants in known disease genes in 17 families (54.8 %) and by novel candidate disease genes in an additional 11 families, making the potential molecular diagnostic rate ~90 %. |
| Eldomery MK, 2017, USA | Suspected monogenic disorders | WES | Outpatient | Mitochondrial DNA sequencing | 74 | < 16 years | Diagnostic yield | The molecular diagnosis was achieved in 30/63 trios (47.6%). Besides this, the analysis workflow yielded evidence for pathogenic variants in disease-associated genes in 4/6 singleton cases (66.6%), 1/1 multiplex family involving three affected siblings, and 3/4 (75%) quartet families. |
| Lionel AC, 2017, Canada | Suspected unspecific genetic disorders | WGS | Outpatient | Standard care | 103 | < 18 years | Diagnostic yeld | WGS identified diagnostic variants in 41% of individuals, representing a significant increase over conventional testing  results (24%; P = 0.01) |
| Meng L, 2017, USA | Suspected monogenic disorders | WES | NICU/PICU | Copy number variation sequencing | 278 | 28 days | Diagnostic yield,  Turnaround time,  Change in clinical management | Overall, molecular diagnosis was achieved in 102/278 infants by clinical exome sequencing with a diagnostic yield of 36.7%. The diagnosis affected medical management in 53/102 (52.0%) of infants. The diagnostic yield, patient age at diagnosis, and medical impact in the group that underwent critical trio exome is significantly different comparing to regular exome testing. |
| Monies D, 2017, Saudi Arabia | Suspected unspecific genetic disorders | WES | Outpatient | Multigene panel test | 270 | < 16 years | Diagnostic yield | Pathogenic or likely pathogenic variants that explain the clinical indications were identified in 34% (27% in panels and 43% in exomes), spanning 279 genes and including 165 novel variants. |
| Tan TY, 2017, Australia | Suspected monogenic disorders | WES | Outpatient | Standard care | 44 | 28 months | Diagnostic yield  Clinical utility  Change in clinical management | Diagnosis was achieved in 23 (52%) by singleton WES. Clinical management was altered in 6 of 23 (26%). |
| Vissers L, 2017, Netherlands | Suspected unspecific genetic disorders | WES | Outpatient | Standard care | 150 | 5 years | Diagnostic yield | WES |
| Du X, 2018, China | Suspected autism Spectrum Disorder | WES | Outpatient | NA | 80 | < 7 years | Diagnostic yield | An overall diagnostic yield of 8.8% was observed in the cohort. |
| Farnaes L, 2018, US | Suspected unspecific genetic disorder | WGS | NICU/PICU | Standard care | 42 | < 1 year | Diagnostic yield, clinical utility, healthcare utilization | WGS had a significantly higher diagnostic sensitivity (43%) than standard of care (10%) |
| Petrikin JE, 2018, USA | Congenital anomalies  Neurological disorders | WGS | NICU/PICU | Standard care | 65 | 4 months | Diagnostic yield | Among infants enrolled in the first 25 days of life, the rate of neonatal diagnosis was higher in cases (32%, 7 of 22) than controls (0%, 0 of 23; difference, 32% [95% CI, 11–53%];p = 0.004). Median age at diagnosis (25 days [range 14–90] in cases vs. 130 days [range 37–451] in controls) and median time to diagnosis (13 days [range 1–84] in cases, vs. 107 days [range 21–429] in controls) were significantly less in cases than controls (p = 0.04). |
| Kingsmore SF, 2019, USA | Suspected unspecific genetic disorders | WGS | NICU, PICU, and CVICU | rWES, rWGS | 213 | ≤ 4 months | Diagnostic yield | The proportion diagnosed by WGS (11 of 24 [46%]) was higher than rWES/rWGS (p¼0.004) and time to result was less (median 4.6 days, p<0.0001). The in-cremental diagnostic yield of reflexing to trio after negative proband analysis was 0.7% (1 of 147). |
| Sanford EF, 2019, US | Suspected unspecific genetic disorders | WGS | PICU | NA | 38 | 5.73 years | Diagnostic yield,  Clinical utility, Change in clinical management | WGS diagnosed a genetic disease in 17 of 38 critically ill children (45%). In four of the 17 patients (24%), the genetic diagnoses led to a change in management while in the PICU |
| Scocchia A, 2019, Mexico | Suspected unspecific genetic disorder | WGS | Outpatient | NA | 60 | 7.6 years | Diagnostic yield  Clinical utility  Change in clinical management | Clinically significant genomic findings were identified in 68.3% (n = 41) of probands. A qualitative assessment of clinical management revealed 48.8% (n = 20) of those diagnosed had a change in clinical course based on their WGS results, despite resource limitations. |
| Qi ZY, 2019, China | Suspected monogenic disorders | WES | NICU | NA | 45 | 2 days | Diagnostic yield | Of the 45 newborns, 12 (27%) were confirmed with monogenic hereditary disorders. The improvement rate of newborns with monogenic hereditary disorders was 67% (8/12) after treatment. |
| Smigiel R, 2020, Poland | Suspected monogenic disorders | WES | PICU | NA | 18 | 8 months | Diagnostic yield | A conclusive genetic diagnosis was obtained in 13 children, corresponding to an overall diagnostic yield of 72.2% |
| Zhang L, 2020, China | epilepsy | WES | Outpatient | NA | 43 | 3.45 years | Diagnostic yield | The overall diagnostic yield was 32.6% |
| Zhu T, 2020, China | Suspected unspecific genetic disorders | WES | NICU | trio-panes and proband panel | 307 | ≤ 100 d | Diagnostic yield | Trio-WES, trio-panel, proband-WES, and proband-panel diagnostic yields were 39.71% (83/209), 68.75% (22/32), 59.09% (26/44), and 33.33% (4/12), respectively. |
| Dimmock D, 2021, USA | Suspected unspecific genetic disorder | WGS | NICU | NA | 184 | < 1 year | Diagnostic yield  Health care resources use | Of 184 infants enrolled, 74 (40%) received a diagnosis by WGS that explained their admission in a median time of 3 days. In 58 (32%) affected individuals, WGS led to changes in medical care. Testing and precision medicine cost $1.7 million and led to $2.2–2.9 million cost savings. |
| Herman I, 2021, USA | Neuromuscular disease | WES | Outpatient | Chromosomal microarray | 106 | < 18 years | Diagnostic yield | A molecular diagnosis was achieved in 37/79 (46%) patients with ES, 4/44 (9%) patients with CMA, and 15/74 (20%) patients with candidate gene testing. |
| Kose M, 2021, Turkey | Suspected mendelian mitochondrial diseases | WES | NR | Targeted Exome Sequencing | 59 | 10 years | Diagnostic yield | 61% of the patients were diagnosed with whole-exome sequencing (WES) (36/59) and 15% with targeted exome sequencing (TES) (9/59). |
| Krantz ID, 2021, US | Suspected unspecific genetic disorder | WGS | Outpatient | delayed WGS  (60 days) | 354 | 15 days | Diagnostic yield  Change in clinical management | At 60 days, twice as many infants in the early group vs the delayed group received a COM (34 of 161 [21.1%; 95% CI, 15.1%-28.2%] vs 17 of 165 [10.3%; 95% CI,6.1%-16.0%];P= .009; odds ratio, 2.3; 95% CI, 1.22-4.32) and a molecular diagnosis (55 of176 [31.0%; 95% CI, 24.5%-38.7%] vs 27 of 178 [15.0%; 95% CI, 10.2%-21.3%];P< .001). At90 days, the delayed group showed a doubling of COM (to 45 of 161 [28.0%; 95% CI,21.2%-35.6%]) and diagnostic efficacy (to 56 of 178 [31.0%; 95% CI, 24.7%-38.8%]). |
| Liu Y, 2021, China | Metabolic disease  Neuromuscular disease  Multiple deformities | WES | PICU | NA | 169 | 10.5 months | Diagnostic yield  Change in clinical management | A total of 43 patients (25%) were diagnosed with monogenic disorders. The results of WES had an impact on the treatment for 30 cases (70%). |
| Liu Y, 2021, China | Developmental disorders | WES or WES+ CNV | Outpatient | Single proband WES vs Trio WES vs WES+CNVs | 94 | 24.7 months | Diagnostic yield  Clinical utility | WES reached a high diagnostic rate (48.7%, 46/94), and de novo (48.3%, 28/58) was the main pathogenic form. The number of uncertain significant locus in the patients taking Trio-WES was significantly lower than that in patients taking Pro-WES (2.1%vs 2.8%). The diagnostic rate of WES accompanied by CNVseq (57.5%, 46/80) was significantly higher (p= 0.016) than WES alone. |
| Ripen AM, 2021, Malaysia | Errors of immunity | WES | Outpatient | Clinical diagnose | 30 | 5 years | Diagnostic yield | Genetic diagnosis was attained in 46.7% (14 of 30) of the patients. Genetic findings differed from the provisional clinical diagnoses in seven cases (50.0%). |
| Scholz T, 2021, Germany | Suspected monogenic disorders | WES | NICU | NA | 61 | 68 days | Diagnostic yield | The overall molecular genetic diagnostic rate within the cohort was 46% (28/61) and 50% (15/30) in the subgroup of preterm neonates. Identifying the genetic cause of disease facilitates individualized management in the majority of patients. |
| Schon KR, 2021, England | Mitochondrial disease | WGS | Outpatients | NA | 345 | 12 years | Diagnostic yield | A definite or probable genetic diagnosis was identified in 98/319 (31%) families, with an additional 6 (2%) possible diagnoses. Of 104 families given a diagnosis, 39 (38%) had a mitochondrial diagnosis and 65 (63%) had a non-mitochondrial diagnosis |
| Sharma S, 2021, US | Hypotonia | whole exome [WES], or whole genome sequencing [WGS] | Outpatient | karyotype, microarray, targeted genetic testing for specific conditions, gene panels | 324 | 5.17 months | Diagnostic yield | Diagnostic yield was 32% for karyotype, 19% for microarray, 30% for targeted genetic tests, 38% for gene panels, and 31% for whole exome sequencing. The combination of microarray and exome sequencing gave the highest diagnostic yield. None of the other tests added significant value in arriving at a diagnosis. |
| Wu B, 2021, China | Neuromuscular,  respiratory, and immunologic/infectious diseases | WGS | Outpatient | trio-rapid genome sequencing | 202 | < 18 years | Diagnostic yield  Change in clinical management | The diagnostic yield of trio-rapid genome sequencing was higher than that of proband-only clinical exome sequencing (36.6% [95% CI, 30.1-43.7%] vs 20.3% [95% CI, 15.1-26.6%], respectively; p = 0.0004), and the average turnaround time for trio-rapid genome sequencing (median: 7 d) was faster than that of proband-only clinical exome sequencing (median: 20 d) (p < 2.2 × 10-16). Sixteen infants (21.6%) experienced a change in clinical management following trio-rapid genome sequencing diagnosis, and 24 infants (32.4%) were referred to a new subspecialist. |
| Leite AJDC, 2022, Brazil | Intellectual Disability (ID), global developmental delay and multiples congenital anomalies | WES. | Outpatient | CMA | 369 | <18 y | Diagnostic yield | CMA was performed in 83 patients who had normal karyotype results resulting in a diagnostic yield of 21.7% (18/83). Exome sequencing with analysis of the ID gene panel was performed in 19 trios of families that had negative results with previous methodologies. With the ID gene panel analysis, mutations were identified in 63.1% (12/19) of the cases. With the three methodologies applied, it was possible to identify the genetic cause of ID in 42.3% (156/369) of the patients. |

**Table S2.** Results of the Quality Assessment of Diagnostic Accuracy Studies -2 (QUADAS-2) tool for studies included in systematic review and meta-analysis, 2015–2022.

| **Study, year** | **RISK OF BIAS** | | | | **APPLICABILITY CONCERNS** | | |
| --- | --- | --- | --- | --- | --- | --- | --- |
|  | **PATIENT SELECTION** | **INDEX TEST** | **REFERENCE STANDARD** | **FLOW AND TIMING** | **PATIENT SELECTION** | **INDEX TEST** | **REFERENCE STANDARD** |
| Taylor JC, 2015 | ☺ | ☺ | ? | ☺ | ☺ | ☺ | ? |
| Zhu X, 2015 | ☺ | ☺ | ☺ | ☺ | ☺ | ☺ | ☺ |
| Farwell, 2015 | ☺ | ☺ | ☺ | ☺ | ☺ | ☺ | ☺ |
| Valencia CA, 2015 | ☺ | ☺ | ? | ☺ | ☺ | ☺ | ? |
| Willig LK, 2015 | ☺ | ☺ | ☺ | ☺ | ☺ | ☺ | ☺ |
| Stavropoulos DJ, 2016 | ☺ | ☺ | ☺ | ☺ | ☺ | ☺ | ☺ |
| Thevenon J, 2016 | ☺ | ☺ | ☺ | ☺ | ☺ | ☺ | ☺ |
| Stark Z, 2016 | ☺ | ☺ | ☺ | ☺ | ☺ | ☺ | ☺ |
| Retterer K, 2016 | ☹ | ☺ | ? | ☺ | ☹ | ☺ | ? |
| Bick D, 2017 | ☹ | ☺ | ? | ☺ | ☹ | ☺ | ? |
| Vissers L, 2017 | ☺ | ☺ | ☺ | ☺ | ☺ | ☺ | ☺ |
| Meng L, 2017 | ☺ | ☺ | ? | ☺ | ☺ | ☺ | ☹ |
| Monies D, 2017 | ☺ | ☺ | ☺ | ☹ | ☺ | ☺ | ☺ |
| Eldomery MK, 2017 | ☹ | ☺ | ? | ☺ | ☹ | ☺ | ? |
| Charng WL, 2017 | ☹ | ☺ | ? | ☺ | ☹ | ☺ | ? |
| Tan TY, 2017 | ☺ | ☺ | ☺ | ☺ | ☺ | ☺ | ☺ |
| Lionel AC, 2017 | ☺ | ☺ | ☺ | ☺ | ☺ | ☺ | ☺ |
| Petrikin JE, 2018 | ☺ | ☺ | ☺ | ☺ | ☺ | ☺ | ☺ |
| Du X, 2018 | ☺ | ☺ | ☺ | ☺ | ☺ | ☺ | ☺ |
| Farnaes L, 2018 | ☺ | ☺ | ☺ | ☹ | ☺ | ☺ | ☺ |
| Qi ZY, 2019 | ☺ | ☺ | ? | ☺ | ☺ | ☺ | ? |
| Kingsmore SF, 2019 | ☺ | ☺ | ? | ☺ | ☺ | ☺ | ? |
| Scocchia A, 2019 | ☺ | ☺ | ☺ | ☺ | ☺ | ☺ | ☺ |
| Sanford EF, 2019 | ☹ | ☺ | ☺ | ☺ | ☹ | ☺ | ☺ |
| Zhu T, 2020 | ☺ | ☺ | ? | ☺ | ☺ | ☺ | ☺ |
| Zhang L, 2020 | ☺ | ☺ | ☺ | ☺ | ☺ | ☺ | ☺ |
| Smigiel R, 2020 | ☺ | ☺ | ☺ | ☺ | ☺ | ☺ | ☺ |
| Liu Y, 2021 | ☺ | ☺ | ? | ☺ | ☺ | ☺ | ? |
| Scholz T, 2021 | ☺ | ☺ | ☺ | ☺ | ☺ | ☺ | ☺ |
| Dimmock D, 2021 | ☺ | ☺ | ? | ☺ | ☺ | ☺ | ? |
| Herman I, 2021 | ☺ | ☺ | ☹ | ☺ | ☺ | ☺ | ☹ |
| Liu Y, 2021 | ☺ | ☺ | ? | ☺ | ☺ | ☺ | ? |
| Kose M, 2021 | ☺ | ☺ | ☺ | ☺ | ☺ | ☺ | ☺ |
| Ripen AM, 2021 | ☺ | ☺ | ☺ | ☺ | ☹ | ☺ | ☺ |
| Wu B, 2021 | ☺ | ☺ | ☺ | ☺ | ☺ | ☺ | ☺ |
| Sharma S, 2021 | ☹ | ☺ | ? | ☺ | ☹ | ☺ | ☺ |
| Krantz ID, 2021 | ☺ | ☺ | ☺ | ☺ | ☺ | ☺ | ☺ |
| Schon KR, 2021 | ☺ | ☺ | ☺ | ☺ | ☹ | ☺ | ☺ |
| Leite AJDC, 2022 | ☺ | ☺ | ? | ☹ | ☹ | ☺ | ☺ |

☺Low Risk ☹High Risk ? Unclear Risk

**Table S3.** GRADE Evidence Profiles for studies included in the systematic review and meta-analysis, 2015–2022.

| Quality assessment | | | | | | Summary of findings | | | | | |
| --- | --- | --- | --- | --- | --- | --- | --- | --- | --- | --- | --- |
|  | | | | | | Number of patients | |  | Absolute risk | |  |
| N. of Studies  (Design) | Limitation | Inconsistency | Indirectness | Imprecision | Other factors | WGS | WES | Relative risk  (95% CI) | Control risk^a^ | Risk difference  (95% CI) | Quality |
|  |  |  |  |  |  |  |  |  |  |  |  |
| Diagnostic Yield  36 (observational) | not serious^b^ | not serious^c^ | not serious^d^ | not serious | none | 444/702 | 1579/3085 | OR 1.24  (1.10–1.40) | 51/100 | 5 more per 100 (2–8) | ⨁⨁⨁◯  Moderate |
|  | | | | | | | | | | | |
| Diagnostic Yield  3 (RCT) | not serious^b^ | not serious^c^ | not serious^d^ | not serious | none | 83/302 | 19/95 | OR 1.54  (1.11–2.12) | 20/100 | 8 more per 100 (2–15) | ⨁⨁⨁⨁  High |

*Abbreviations:* GRADE, Grading of Recommendations Assessment, Development, and Evaluation; WES, Whole exome sequencing; WGS, Whole genome sequencing; RCT, randomized controlled trial; CI, confidence interval; OR, odds ratio.

^a^ The control rate is based on the median control group risk across studies.

^b^ See risk of bias assessment in Table 1 and Figure 2.

^c^ Studies all demonstrated consistency in direction of effect.

^d^ Diagnostic Yield is directly reported by included studies.

**Table S4.** Summary of findings table for studies included in the systematic review and meta-analysis, 2015–2022.

| **Whole genome sequencing compared with Whole exome sequencing for pediatric population with suspected genetic disorders** | | | | | | |
| --- | --- | --- | --- | --- | --- | --- |
| Population: Pediatric patients with suspected genetic disorders  Intervention: Whole genome sequencing  Comparison: Whole exome sequencing | | | | | | |
|  | Estimated risks (95% CI) | | | | | |
|  | Control risk^a^ | Intervention risk |  | | | |
| Outcomes | WES | WGS | Relative effects (95% CI) | N. of participants (studies) | Quality of the evidence (GRADE) | Comments |
|  |  |  |  |  |  |  |
| Diagnostic Yield  36 (observational) | 51 per 100 | 57 per 100 (54–59) | OR 1.24 (1.10–1.40) | 3787 (36) | ⨁⨁⨁◯  Moderate | Whole genome sequencing may result in a slight increase in diagnostic yield. |
|  | | | | | | |
| Diagnostic Yield  3 (RCT) | 20 per 100 | 28 per 100 (22–35) | OR 1.54 (1.11–2.12) | 397 (5) | ⨁⨁⨁⨁  High | Whole genome sequencing results in a slight increase in diagnostic yield. |

*Abbreviations*: GRADE, Grading of Recommendations Assessment, Development, and Evaluation; WES, Whole exome sequencing; WGS, whole genome sequencing; RCT, randomized controlled trial; CI, confidence interval; OR, odds ratio.

^a^ The basis for the control risk is the median control group risk across studies. The intervention risk (and its 95% CI) is based on the control risk in the comparison group and the relative effect of the intervention (and its 95% CI).

**Table S5.** Meta-regression analysis considering studies with lower diagnostic yield (i.e., inferior to the pooled estimate for each diagnostic technique).

|  | **Adjusted Beta** | **Odds Ratio [95% CI]** | ***p*-value** |
| --- | --- | --- | --- |
| Technique (reference = *WES*) |  |  |  |
| *UC* | -2.229 | 0.11 [0.06 - 0.18] | <0.0001 |
| *WGS* | 0.185 | 1.20 [0.94 - 1.54] | 0.1414 |
| Monogenic disease, *yes* | -0.032 | 0.97 [0.73 - 1.29] | 0.8290 |
| NICU/PICU setting, *yes* | -0.038 | 0.96 [0.75 - 1.24] | 0.7641 |
| Publication date, *after 2017* (reference = *before 2017*) | -0.103 | 0.90 [0.69 - 1.17] | 0.4371 |

Abbreviations: CI, confidence interval; WES, Whole exome sequencing; WGS, Whole genome sequencing; NICU, neonatal intensive care unit; PICU, paediatric intensive care unit; UC, usual care.

**Table S6.** Meta-regression analysis considering studies with higher diagnostic yield (i.e., superior to the pooled estimate for each diagnostic technique).

|  | **Adjusted Beta** | **Odds Ratio [95% CI]** | ***p*-value** |
| --- | --- | --- | --- |
| Technique (reference = *WES*) |  |  |  |
| *UC* | -1.404 | 0.25 [0.18 - 0.33] | <0.0001 |
| *WGS* | 0.143 | 1.15 [0.91 - 1.47] | 0.2417 |
| Monogenic disease, *yes* | 0.434 | 1.54 [1.21 - 1.97] | 0.0004 |
| NICU/PICU setting, *yes* | -0.253 | 0.78 [0.63 - 0.96] | 0.0206 |
| Publication date, *after 2017* (reference = *before 2017*) | 0.128 | 1.14 [0.79 - 1.63] | 0.4859 |

Abbreviations: CI, confidence interval; WES, Whole exome sequencing; WGS, Whole genome sequencing; NICU, neonatal intensive care unit; PICU, paediatric intensive care unit; UC, usual care.
